# Identification and Characterization of Three Novel Small Interference RNAs That Effectively Down-Regulate the Isolated Nucleocapsid Gene Expression of SARS Coronavirus

**DOI:** 10.3390/molecules16021544

**Published:** 2011-02-11

**Authors:** Ying-Li Cao, Ying Wang, Rong Guo, Fan Yang, Yun Zhang, Shu-Hui Wang, Li Liu

**Affiliations:** Department of Microbiology, Institute of Basic Medical Sciences, Chinese Academy of Medical Sciences & School of Basic Medicine, Peking Union Medical College, Beijing 100005, China

**Keywords:** siRNA, SARS-CoV, nucleocapsid gene, RT-PCR, EGFP

## Abstract

Nucleocapsid (N) protein of severe acute respiratory syndrome-associated coronavirus (SARS-CoV) is a major pathological determinant in the host that may cause host cell apoptosis, upregulate the proinflammatory cytokine production, and block innate immune responses. Therefore, N gene has long been thought an ideal target for the design of small interference RNA (siRNA). siRNA is a class of small non-coding RNAs with a size of 21-25nt that functions post-transcriptionally to block targeted gene expression. In this study, we analyzed the N gene coding sequences derived from 16 different isolates, and found that nucleotide deletions and substitutions are mainly located at the first 440nt sequence. Combining previous reports and the above sequence information, we create three novel siRNAs that specifically target the conserved and unexploited regions in the N gene. We show that these siRNAs could effectively and specifically block the isolated N gene expression in mammal cells. Furthermore, we provide evidence to show that N gene can effectively up-regulate M gene mediated interferon β (IFNβ) production, while blocking N gene expression by specific siRNA significantly reduces IFNβ gene expression. Our data indicate that the inhibitory effect of siRNA on the isolated N gene expression might be influenced by the sequence context around the targeted sites.

## 1. Introduction 

Severe acute respiratory syndrome (SARS) that spread worldwide in 2003 is caused by a novel type of coronavirus called SARS-associated coronavirus (SARS-CoV). SARS-CoV is an enveloped and positively single-stranded RNA virus with a typical genome size ~29.7 kb [1,2,3]. The viral envelope is a lipid bilayer enbedded with three viral transmembrane proteins: the matrix (M), envelope (E) and spike (S) proteins. The genomic RNA of SARS-CoV is protected and packaged by the viral nucleocapsid (N) protein by forming a helical structure of ribonucleoprotein complex within the viral envelope. In addition, the physical interaction between the N and M proteins might be necessary for the assembly of coronavirus [4,5,6]. 

The N gene of SARS-CoV is located at a region proximal to the 3’ end of the viral genome with a size of 1269nt long that encodes for a 422aa basic protein. The main functions of N protein are two folds: 1) it binds to viral genomic RNA for the formation of viral nucleocapsid; 2) it also self-associates into polymer that might be critical for the helical structure formation [7]. Protein structural study reveals that the SARS-CoV N protein contains two structural domains flanked by intrinsically disordered (ID) regions [8]. The N terminal domain (NTD, amino acid residues 45–181) can bind non-specifically to a variety of nucleotide substrates [9] and functions as a putative RNA binding domain associated with the viral RNA genome [10], whereas the C terminal domain (CTD, amino acid residues 248–365) is thought to be responsible for the dimerized self-association [8,11]. However, recent studies show that the C terminal sequence of N protein also promotes higher ordered oligomerization and is able to interact with nucleic acids as well [7,12]. Moreover, Chang demonstrated that all ID sequences are able to bind RNA [13], indicating that the multisite nucleic acid binding property of SARS-CoV N protein may have an inherent advantage to promote the formation of viral helical nucleocapsid core. 

SARS-CoV infection is a life-threatening disease that often develops with acute lung injury and acute respiratory distress syndrome. The inflammation and immune responses in the host are often induced or damaged by viral gene products such as N gene products. SARS-CoV N protein possesses multifarious activities and may be actively involved in SARS-CoV induced pathogenesis [14]. For instance, N protein induces the pro-inflammatory responses by activating the promoter activity of either cyclooxygenase-2 (COX-2) or IL-6 by directly interacting with the NFκB binding element [15,16].

Overexpression of N protein in serum starved cell lines such as monkey kidney Cos-1 cells [17] or human pulmonary fibroblast HPF cells [18] induces apoptosis. More importantly, SARS-CoV N protein can function as an antagonist to counteract the host innate immune response by inhibiting the activity of IRF3 and NFkB, and subsequently blocking interferon β production [19].

SARS-CoV N protein is believed to be one of the major pathological determinants in the host cells [14]. Therefore, down-regulating N protein expression by the small interference RNA (siRNA) approach might be good strategy to reverse the virus-induced damage to the host and may help development of a more effective therapeutic means to control viral transmission. siRNA is a type of RNA molecule with 21-25nt in length that functions post-transcriptionally to down-regulate the targeted gene expression. SARS-CoV N gene specific siRNAs have been designed and investigated by a number of groups, and at least 23 targeted sites of N gene specific siRNAs have been selected and tested [20,21,22,23,24]. Sequence analysis indicates that all these known siRNAs are located at 2/3 of the 5’ N gene sequence (within first 860nt out of the total 1269nt). Nucleotide substitution and deletion mutations can be frequently recovered from the first 440nt sequence of N gene. Therefore, these sequence alterations may have a potential to weaken the effect of siRNA mediated gene silencing. In the current study, we compared the N gene sequences derived from 16 different isolates of SARS-CoV and selected three novel siRNA targeting sites in the N gene, including one targeting the 3’ terminus of the gene. Functional analysis indicates that all three novel siRNAs are effective in downregulating N gene expression. Moreover, our study demonstrates that SARS-CoV N gene is able to up-regulate the M gene mediated interferon β production, while the N gene specific siRNA can effectively reduce this up-regulation.

## 2. Results and Discussion 

### 2.1. Identification of three novel targeted sites for N gene specific siRNAs

**Figure 1:**
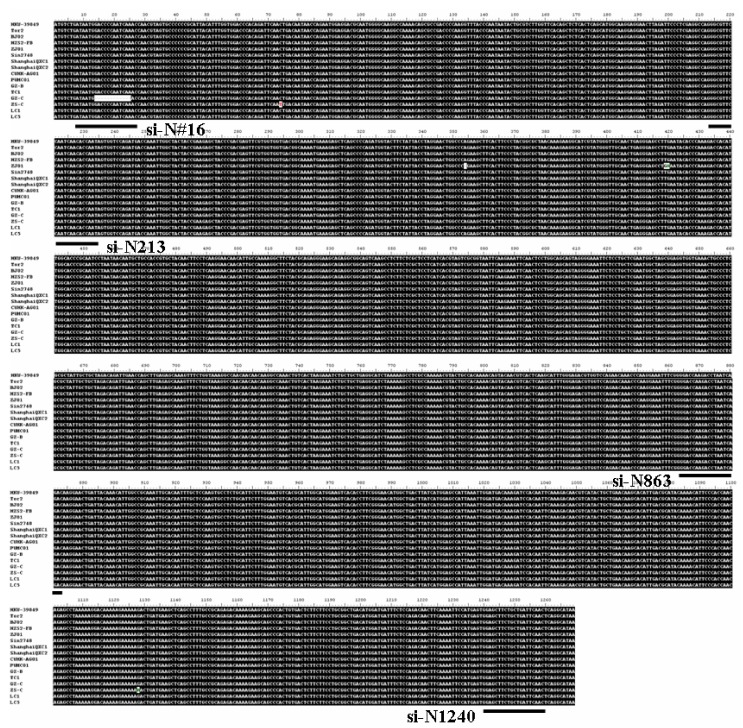
Sequence alignment of the N genes derived from 16 different SARS-CoV isolates.

SARS-CoV N protein is a multi-functional protein that contributes greatly to viral induced pathogenesis and often serves as the therapeutic targeting site for the development of anti-viral drugs against SARS-CoV infection, including siRNA. SARS-CoV is a positive single-stranded RNA virus frequently associated with sequence alterations during viral transmission. A better siRNA should be designed to target the conserved region in the targeted sequence. To this end, we randomly selected the N genes derived from 16 different isolates of SARS-CoV: HKU-39849 (AY278491), Tor2 (AY274119), BJ02 (AY278487), HZS2-FB (AY394987), ZJ01 (AY297028), Sin2748 (AY283797), ShanghaiQXC1 (AY463059), ShanghaiQXC2(AY463060), CUHK-AG01 (AY345986), PUMC01 (AY350750), GZ-B (AY394978), TC1 (AY338174), GZ-C (AY394979), ZS-C (AY95003), LC1 (AY394998.1) and LC5(AY395002.1). ClustalW analysis revealed that a total of five sequence alterations (two sequence deletions and three nucleotide substitutions) occurred in the N gene in these isolates (Figure 1). Interestingly, sequence alterations in the N gene are frequently uncovered in the 5’ portion of the gene (within the first 440nt), which is similar to our previous observation on SARS-CoV M gene [25]. However, different from the M gene mutation, which is associated with single nucleotide substitution, the N gene mutation tends to be larger in size, such as twelve nucleotide deletions (nt14-25) and di-nucleotide substitutions (nt419-420, Figure 1). The N gene also possesses two additional single nucleotide substitutions (nt74 and nt1128) and one single nucleotide deletion (nt384). 

Previously, a number of N gene-specific siRNAs based on random selection have been reported by other groups [20,21,22,23,24]. Sequence analysis revealed that all these siRNAs are located within the first 859nt sequence of the N gene, and no targeted site has been selected for the last 410nt sequence. Considering the nucleotide substitutions in N gene as well as the previous reports, we chose three unexploited regions that were well conserved in the N genes among the 16 isolates of SARS-CoV. The selected targeted sites were at +213~+233nt, +863~+883nt and +1240~+1260nt relative to the 5’ ATG initiation codon, and their respective siRNAs were named as si-N213, si-N863 and si-N1240 (Figure 1). A known siRNA (named si-N#16, previous name No.16 in reference [21]) that targeted the 5’ terminus (+7~+27nt) of N gene was also constructed for control purposes. 

### 2.2. Expression analysis of N gene in mammal cells

**Figure 2:**
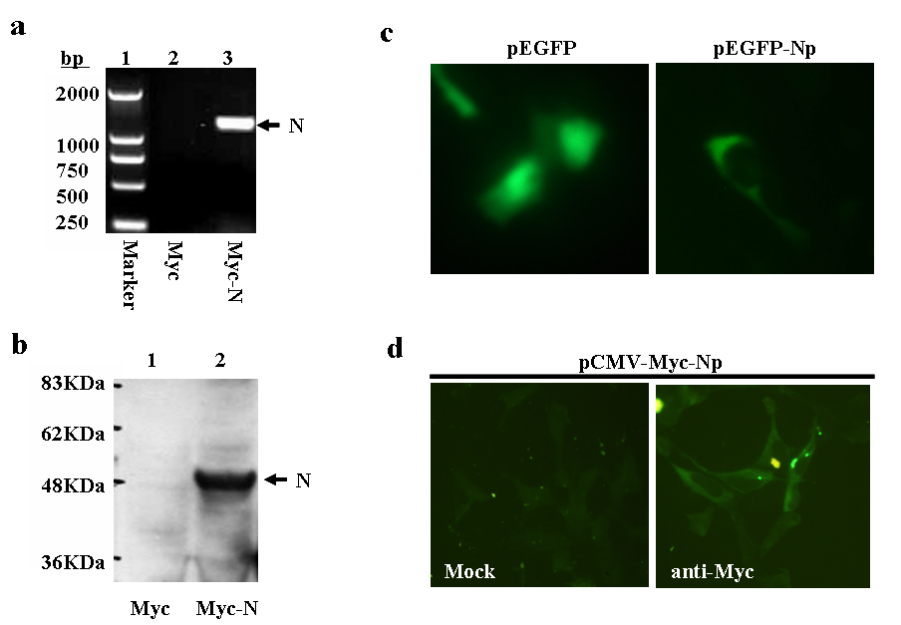
Expression analysis of the isolated SARS-CoV N gene.(a) RT-PCR analysis on N gene expression. Total RNAs were isolated from pCMV-Myc (lane 2) or pCMV-Myc-Np (lane 3) transfected HEK293 cells and subjected RT-PCR. (b) Western blot analysis on N gene expression in HEK293 cells. HEK293 cells were transfected with either pCMV-Myc or pCMV-Myc-Np. After 48 h, the transfected cells were lysed. Equal amount of cell lysates were resolved onto 12% SDS-PAGE. The reaction products were probed with anti-Myc antibody. (c) Analysis on EGFP-N gene expression by fluorescence microscope. HEK293 cells were transfected with the either pEGFP-C or pEGFP-Np. (d) Cellular localization of N protein by immunostaining approach. HEK293 cells were transfected with pCMV-Myc-Np. The transfected cells were probed with either PBS (mock) or anti-Myc antibody as primary antibody.

The full length of N gene cDNA was amplified from SARS-CoV strain HKU-39849 infected Vero E6 cells. After confirmation by sequencing analysis, the N gene was then subcloned into eukaryotic expression vector pCMV-Myc to generate a pCMV-Myc-Np plasmid construct. The expression of N gene was confirmed by RT-PCR and Western blot analysis (Figure 2a and 2b). To visualize the subcellular localization of N protein, N gene was also fused with EGFP to make a pEGFP-Np fusion gene construct. After transfection into HEK293 cells, EGFP-Np fusion protein was mainly distributed in the cytoplasm (Figure 2c). Alternatively, HEK293 cells transfected with pCMV-Myc-Np were subjected to immunostaining analysis. In agreement with the above result, as well as a previous report [14], the Myc-tagged Np gene products were indeed predominantly expressed in the cytoplasm (Figure 2d). 

### 2.3. Three novel siRNAs specifically and effectively inhibits the isolated SARS-CoV N gene expression

**Figure 3:**
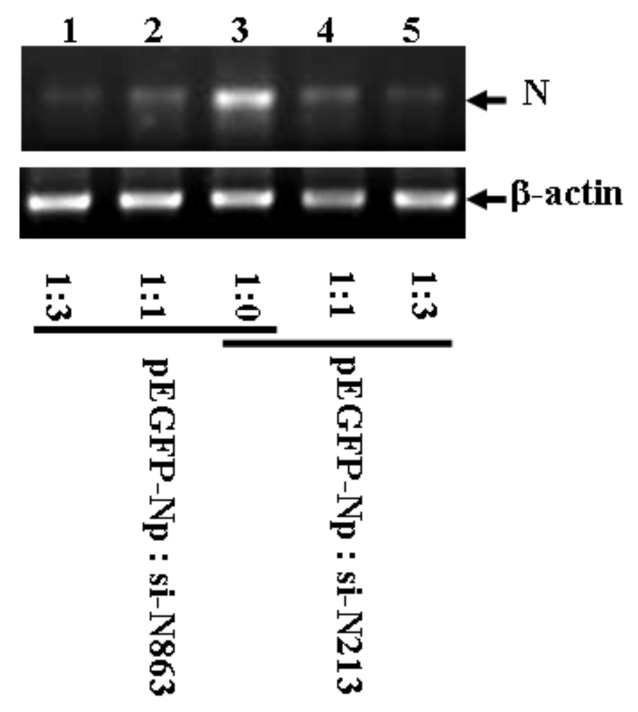
RT-PCR analysis on siRNA mediated N gene inhibition.

To test if the selected siRNAs have an inhibitory effect on N gene expression, pEGFP-Np was co-transfected with increased doses of either si-N213 or si-N863 into HEK293 cells. Figure 3 demonstrates that both si-N213 and si-N863 inhibited pEGFP-Np expression post-transcriptionally and functioned in a dose-dependent manner, indicating that both siRNAs are effective in inhibiting the targeted mRNA expression. 

**Figure 4:**
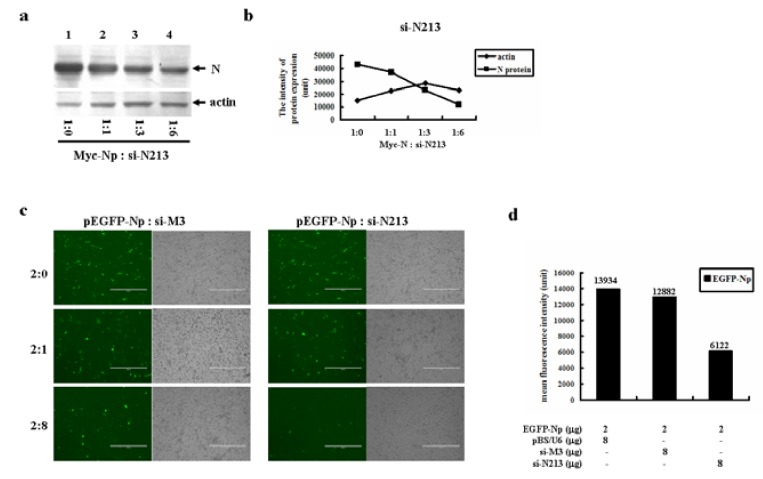
The inhibitory effect of si-N213 on the isolated SARS-CoV N gene expression. (a) Western blot analysis on the effect of si-N213 on N gene expression. pCMV-Myc-Np was co-transfected with the increased doses of pBS/U6-si-N213 into HEK293 cells. After 48 h, the transfected cells were lysed. Equal amount of cell lysates were resolved onto 12% SDS-PAGE. The reaction products were probed with anti-Myc or anti-actin antibodies. (b) Quantitation of the individual band intensity detected in (a). (c) si-N213 but not si-M3 effectively inhibited EGFP-N gene expression. About 2 μg of pEGFP-N plasmid was co-transfected with increased doses of either si-M3 or si-N213. (d) The mean fluorescence intensity (MFI) of EGFP-Np fusion gene expression in the absence or presence of si-N213 was detected by flow cytometric analysis. SARS-CoV M gene specific siRNA, si-M3, was served as a non-specific control.

The effect of siRNA on N gene repression was further confirmed by Western blot analysis. Figure 4a demonstrates a significant reduction in N protein expression as the ratio of N to si-N213 increased. 

Quantitation of the band intensity revealed about 4 fold reduction in N protein expression when co-transfection with higher doses of si-N213 (Figure 4b). The specificity of si-N213 on N gene expression was further confirmed by using a non-specific siRNA, si-M3, as a negative control. si-M3 has been shown to be a potent inhibitor to SARS-CoV M gene expression [25,26]. Higher doses of si-N213 but not si-M3 dramatically inhibited EGFP-Np gene expression, indicating the specificity of si-N213 mediated N gene repression (Figure 4c). Quantitative analysis by flow cytometric approach further demonstrated that si-N213 could specifically and markedly reduce EGFP-Np gene expression (Figure 4d). 

**Figure 5:**
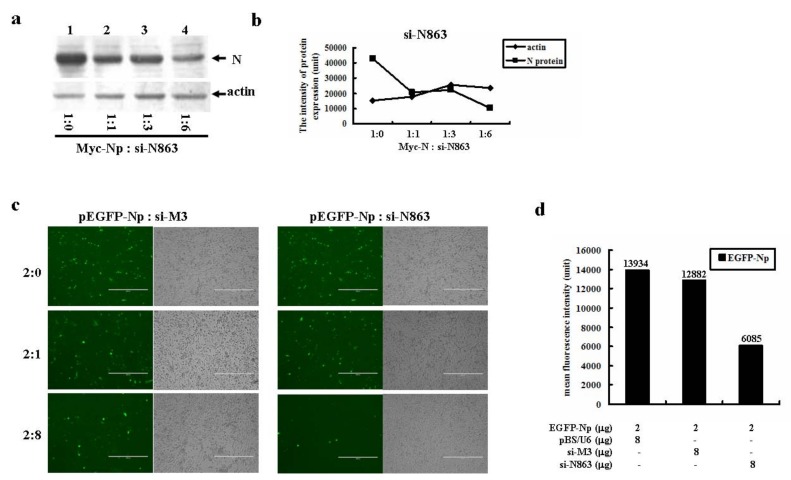
The inhibitory effect of si-N863 on the isolated SARS-CoV N gene expression. (a) Western blot analysis on the effect of si-N863 on N gene expression. pCMV-Myc-Np was co-transfected with the increased doses of pBS/U6-si-N863 into HEK293 cells. After 48 h, the transfected cells were lysed. Equal amount of cell lysates were resolved onto 12% SDS-PAGE. The reaction products were probed with anti-Myc or anti-actin antibodies. (b) Quantitation of the individual band intensity detected in (a). (c) si-N863 but not si-M3 effectively inhibited EGFP-N gene expression. About 2 μg of pEGFP-Np plasmid was co-transfected with increased doses of either si-M3 or si-N863. (d) The mean fluorescence intensity (MFI) of EGFP-Np fusion gene expression in the absence or presence of si-N863 was detected by flow cytometric analysis. SARS-CoV M gene specific siRNA, si-M3, was served as a non-specific control.

Similarly, si-N863, which targeted at the 3’ half of N gene, also dramatically inhibited N protein expression by about four-fold when the molar ratio of si-N863: N reached 6:1 (Figure 5a and 5b). The specificity of si-N863 mediated N gene repression was demonstrated by the fact that higher doses of si-N863, but not si-M3, dramatically inhibited EGFP-Np gene expression (Figure 5c). The results were further confirmed by measuring the mean fluorescent intensity (MFI) of the transfected cells co-transfected with EGFP-Np and the indicated siRNAs (Figure 5d). 

**Figure 6:**
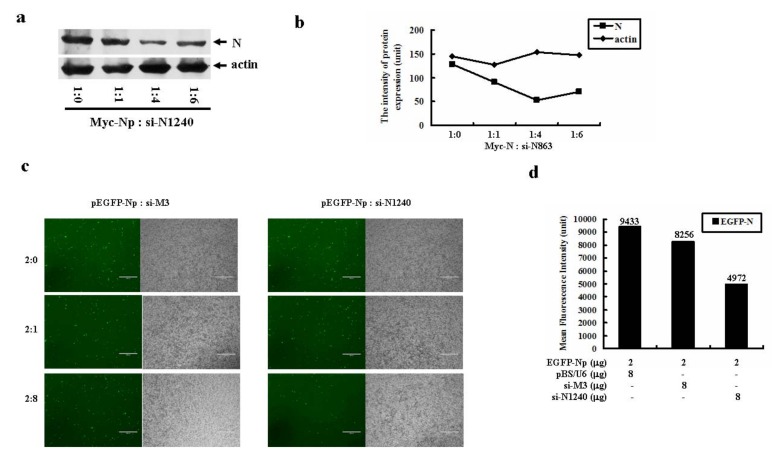
The inhibitory effect of si-N1240 on the isolated SARS-CoV N gene expression. (a) Western blot analysis on the effect of si-N1240 on N gene expression. pCMV-Myc-Np was co-transfected with the increased doses of pBS/U6-si-N1240 into HEK293 cells. After 48 h, the transfected cells were lysed. Equal amount of cell lysates were resolved onto 12% SDS-PAGE. The reaction products were probed with anti-Myc or anti-actin antibodies. (b) Quantitation of the individual band intensity detected in (a). (c) si-N1240 but not si-M3 effectively inhibited EGFP-N gene expression. About 2 μg of pEGFP-Np plasmid was co-transfected with increased doses of either si-M3 or si-N1240. (d) The mean fluorescence intensity (MFI) of EGFP-Np fusion gene expression in the absence or presence of si-N1240 was detected by flow cytometric analysis. SARS-CoV M gene specific siRNA, si-M3, was served as a non-specific control.

Sequence analysis reveals that all the N gene siRNAs reported by other groups have their own recognition sites, mainly located within the first 859nt sequence of the N gene [20,21,22,23,24]. Therefore, to our knowledge, the last 410nt sequence of N gene has not been designed for siRNA targeting. To this end, we created the third siRNA that targets to the 3’ terminus (+1240~+1260nt) of N gene, and named it si-N1240. Western blot analysis demonstrated that si-N1240 markedly inhibited Myc-tagged N protein expression in a dose dependent manner (Figure 6a and 6b). In addition, pEGFP-Np was also co-transfected with increased doses of either si-M3 or si-N1240 into targeted cells. The result shown in Figure 6c clearly demonstrated that higher doses of si-N1240, but not si-M3, dramatically inhibited EGFP-Np gene expression. The result was further confirmed by flow cytometric analysis by measuring the mean fluorescent intensity (MFI) of the transfected cells co-transfected with EGFP-Np and the indicated siRNAs (Figure 6d). 

Overall, the above results provide strong evidence to show that all three novel siRNAs (si-N213, si-N863 and si-N1240) are specific and effective inhibitors to block the isolated SARS-CoV N gene expression. 

### 2.4. The strength of si-N213, si-N863 and si-N1240 on the isolated SARS-CoV N gene expression 

**Figure 7:**
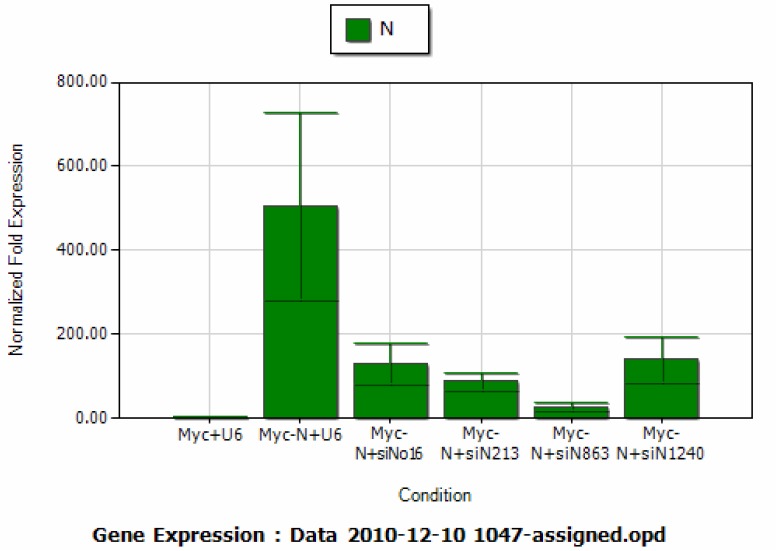
Real time quantitative RT-PCR (qRT-PCR) analysis on the inhibitory effect induced by N gene specific siRNA.

To assess the strength of si-N213, si-N863 and si-N1240 mediated N gene repression, we chose a known siRNA, si-N#16 (=No. 16), which has been shown to be a potent N gene inhibitor [21] as a positive control. About 1.4 μg of pCMV-Myc-Np was co-transfected with 4.2 μg of each plasmid pBS/U6, si-N#16, si-N213, si-N863 and si-N1240 into targeted cells. The real time qRT-PCR result showed that si-N#16, si-N213, si-N863 and si-N1240 all induced significant inhibition on the isolated N gene expression as compared with that of the vector control (Figure 7). The quantitative analysis showed that the N gene inhibitions induced by si-N#16, si-N213, si-N863 and si-N1240 were about 4.0, 5.8, 19.6 and 3.6-fold, respectively (Figure 7), indicating that si-N863 might be a more potent inhibitor on N gene expression. 

### 2.5. N gene specific siRNA effectively reduces N gene mediated interferon β production

**Figure 8:**
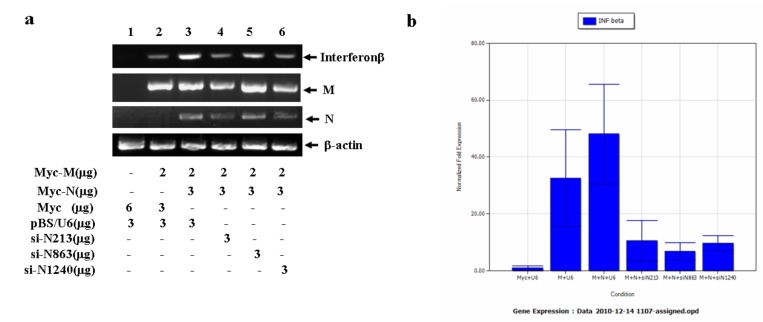
The inhibitory effect of N gene siRNAs on N gene mediated up-regulation of interferon β production.

Previously, we demonstrated that SARS-CoV M gene could upregulate INFβ gene expression in a transient transfection system [25]. Also, it has been shown that INFβ gene expression and transcription could be inhibited by SARS-CoV infection, probably due to the presence of N gene products [27]. To detect the influence of N gene on M mediated INFβ production, N and M were co-transfected into HEK293 cells. Interestingly, we found that the N gene was not able to inhibit, but rather enhanced M gene mediated INFβ mRNA production (Figure 8a). Addition of si-N213, si-N863 or si-N1240 in the co-transfection system significantly reduced INFβ mRNA production (Figure 8a). The semi-quantitative RT-PCR result was further confirmed by using real time qRT-PCR analysis (Figure 8b), indicating that the identified N gene specific siRNA could functionally counteract the N gene mediated cellular processes.

## 3. Experimental 

### 3.1. Cell lines and reagents

Human embryonic kidney cell line 293 (HEK293) cells were obtained from the Cell Culture Center of Institute of Basic Medical Sciences, Chinese Academy of Medical Sciences. African green monkey kidney epithelial cell line Vero E6 was provided by Dr. K.Y. Yuen from the University of Hong Kong. Cells were cultured in Dulbecco's Modified Eagle Medium (HyClone, South Logan, UT, USA) supplemented with 10% fetal calf serum and incubated in a 37 °C incubator containing 5% CO_2_. Anti-Myc and anti-actin antibodies were purchased from Santa Cruz Biotechnology (Santa Cruz, CA, USA). Horseradish peroxidase (HRP)-labeled goat anti mouse IgG and enhanced chemiluminescence (ECL) detection kit were also derived from Santa Cruz Biotechnology. 

### 3.2. Plasmid construction

The SARS-CoV N gene was isolated from SARS-CoV strain HKU-39849 [28] (provided by Dr. KY Yuen, The University of Hong Kong) by standard RT-PCR with a pair of primers: N5:5′-tatagaatt ctgtctgataatggaccccaat-3′ and N3:5′-tataggtaccttatgcctgagttgaatcag-3′. The amplified full length of the N gene was first subcloned into pGEM-T easy vector. After confirmation by sequencing analysis, the N gene products were released by EcoRI/KpnI double digestion and then subcloned into the respective sites of pCMV-Myc to generate pCMV-Myc-N. The N gene products were also inserted into the EcoRI/KpnI sites of pEGFP-N1 vector to form pEGFP-Np fusion gene construct. 

For construction of N gene specific siRNAs, three novel targeted sites (213-233nt, 863-883nt and 1240-1260nt) in the N gene coding sequence were selected. Two oligos for each targeted site were synthesized as siN-213-F, 5’-gggcgttccaatcaacaccaaaagcttttggtgttgattggaacgccctttttg-3’ and siN-213-Re, 5’-aattcaaaaagggcgttccaatcaacaccaaaagcttttggtgttgattggaacgccc-3’; siN-863-F, 5′-gggaccaagacctaa tcagacaagcttgtctgattaggtcttggtccctttttg-3′ and siN-863-Re, 5′-aattcaaaaagggaccaagacctaatcagacaagcttgt ctgattaggtcttggtccc-3′; siN-1240-F, 5′-ggagcttctgctgattcaactaagcttagttgaatcagcagaagctcctttttg-3′ and siN-1240-Re, 5′-aattcaaaaaggagcttctgctgattcaactaagcttagttgaatcagcagaagctcc-3′.

The oligos siN-213-F and siN-213-Re, siN-863-F and siN-863-Re, siN-1240-F and siN-1240-Re were annealed pair-wisely to form duplexes. To construct the siRNA targeting to the 5′ terminus of N gene (named as si-No16 that targets to 7-27nt) as described by Shi *et al.* [21], two synthesized oligos 5′-gataatggaccccaatcaaacaagcttgtttgattggggtccattatctttttg-3′ and 5′-aattcaaaaagataatggaccccaatcaaacaa gcttgtttgattggggtccattatc-3′ were also annealed. The duplex products were then subcloned into pBS/U6 [29] (provided by Dr. Yang Shi, Harvard Medical School) to form pBS/U6-siN213, pBS/U6-siN863, pBS/U6-siN1240 and pBS/U6-No16, respectively.

### 3.3. Reverse transcription-polymerase chain reaction (RT-PCR) and real time quantitative RT-PCR (qRT-PCR)

Total RNAs were extracted from the cultured cells with TRIzol (Invitrogen, Carlsbad, CA). All primers used in the RT-PCR reactions were listed in Table 1. One μg of total RNAs was first reverse transcribed using AMV reverse transcriptase (Promega, USA). About 2 μL of the transcribed cDNAs was subjected to standard PCR reaction using N gene specific primers. One-step real-time quantitative RT-PCR (qRT-PCR) (Takara Biotechnology, Dalian, China) was also performed to monitor the targeted gene expression. Real time qRT-PCR was carried out with iQ5 real-time PCR detection system (Bio-Rad Laboratories) at the following conditions: 42 °C for 5min and 95 °C for 10sec; 95 °C for 5 sec and 60 °C for 10 sec and repeated for 40 cycles. The dissociation of the reaction products was conducted from 55 °C to 95 °C as the temperature rose at 0.2 °C per ten seconds.

### 3.4. Transient transfection

Cell cultured in 35-mm dishes were transiently transfected with the indicated plasmid DNAs using ProFection^®^ Mammalian Transfection Systems (Promega, USA) according to the manual instruction. Briefly, transfected DNAs were first mixed with 37 μL of 2M CaCl_2_ and brought to total 300 μL with sterile and deionized water. Then the DNA-CaCl_2_ mixture was added into equal volume of 2×HBS drop by drop accompanying with gentle vortexing. After 15 minutes incubation, the reaction mixture was evenly distributed into the cell culture medium and incubated for 48 hours before harvesting.

### 3.5. Western blot analysis

The transfected cells were lysed with a lysis buffer containing 1% NP-40, 50 mM Tris-HCl (pH 7.5), 120 mM NaCl, 200 μM NaVO_4_, 1 μg/mL leupeptin, 1 μg/mL aprotinin, and 1 μM PMSF. About 15 μg of cell lysate for each sample was resolved onto 12% SDS-PAGE. After separation, the separated proteins were transferred onto Hybond nitrocellular membrane (Pharmacia). The transferred membrane was first probed with a primary antibody. Then, a secondary antibody labeled with horseradish peroxidase was added to the reaction and finally visualized with an ECL kit.

## 4. Conclusions 

SARS-CoV N gene has long been selected as one of the major targets for siRNA design. However, genomic alterations such as nucleotide deletion and substitution frequently occur in SARS-CoV. This type of change has the potential to weaken the inhibitory effect induced by siRNA. In the current study we analyzed the N gene sequences derived from 16 different isolates and found that in addition to single nucleotide substitutions, the N gene also possesses a longer nucleotide deletion and di-nucleotide substitution. Previous studies on M gene specific siRNAs indicate that the siRNA targeting site and its surrounding sequences may influence the inhibitory effect [25]. The current study further support this observation by showing that si-N#16, which targets a twelve nucleotide deletion region in the N gene of one viral strain generates a less inhibitory effect than that of either si-N213 or si-N863 (Figure 7b). Although N gene specific siRNA has been intensively studied, no siRNA was reported for the last 410nt sequence. In this study, we created and tested a third siRNA (si-N1240) which was effective to target the 3’ terminal sequence of N gene and subsequently in downregulating N gene expression. Interestingly, the inhibitory effect induced by si-N1240 was similar to that of si-N#16, implying that siRNAs targeting at both the 5′ and 3′ terminal sequences of the isolated N gene might induce less inhibition. We also notice that there might be a discrepancy between our study and a previous report by Shi *et al*. in which they demonstrated that si-N#16 (No.16) was the strongest inhibitor among the eleven N gene siRNAs tested [21]. This discrepancy might be due to: 1) the potential structural difference between vector expressed N mRNA and virus derived subgenomic N mRNAs and/or 2) the differences in the expression system used by us (pBS/U6) versus Shi’s (chemical synthesized siRNAs). Finally, we provide evidence to show that SARS-CoV N gene products were able to up-regulate M gene mediated INFβ production, while N gene specific siRNA could functionally reduce this enhancement. However, the mechanism underlining N gene mediated INFβ production remains an interesting point to be further addressed. 

## Figures and Tables

Three novel siRNAs (si-N213, si-N863 and si-N1240) targeting at 213~233nt, 863~883nt and 1240~1260nt, respectively, are underlined. A known si-N#16 (No.16) targeting at 7~27nt reported by Shi *et al.* [21] is also indicated.

pEGFP-Np was co-transfected with the increased dose of either pBS/U6-si-N213 or pBS/U6-si-N863 into HEK293 cells. Total RNAs were isolated from the transfected cells and subjected to standard RT-PCR by using N specific primers. Beta actin expression was served as internal control.

About 1.4 μg of pCMV-Myc-Np was co-transfected with 4.2 μg of each plasmid si-N#16, si-N213, si-N863 and si-N1240. Total RNAs were isolated from the transfected cells and subjected to qRT-PCR analysis. Both β-actin-R and SARS-N-R primers were used in this analysis. The strength of specific siRNA on N gene repression relative to the negative control (pBS/U6) was indicated.

pCMV-Myc-M was co-transfected with pCMV-Myc-Np in the presence or absence of N gene specific siRNAs (pBS/U6-si-N213, pBS/U6-si-N863 and pBS/U6-si-N1240) into HEK293 cells. (a) About 1 μg of total RNA isolated from each reaction was subjected to RT-PCR analysis with IFNβ, M. N or β-actin specific primers. (b) qRT-PCR analysis on the inhibitory effect on interferon β production induced by N gene specific siRNAs.

**Table 1:** Primers used in RT-PCR analysis.

Gene name	GenBank ID	Forward primer	Reverse primer	Size of product (bp)
*β-actin* ^†^	BC009275	5′-cacactgtgcccatctacga-3′	5′-ctgcttgctgatccacatct-3′	600
*INFβ*	NM_002176	5′-atgaccaacaagtgtctcct-3′	5′-ttcagtttcggaggtaacct-3′	564
*SARS-N* ^†^	AY278491	5′-atgtctgataatggacccca-3	5′-tactgctgccaggagttgaa-3′	610
*β-actin-R* ^††^	BC009275	5′-tccatcatgaagtgtgacgt-3′	5′-ctcaggaggagcaatgatct-3′	161
*SARS-N-R* ^††^	AY278491	5′-atgtctgataatggacccca-3′	5′-atgctgagtgagagctgtga-3′	180

^†^ primers for standard RT-PCR; ^††^ primers for quantitative RT-PCR.
